# *LMO1* Gene Polymorphisms Reduce Neuroblastoma Risk in Eastern Chinese Children: A Three-Center Case-Control Study

**DOI:** 10.3389/fonc.2018.00468

**Published:** 2018-10-23

**Authors:** Lili He, Jinhong Zhu, Fei Han, Yingzi Tang, Chunlei Zhou, Jincheng Dai, Yizhen Wang, Haixia Zhou, Jing He, Haiyan Wu

**Affiliations:** ^1^Department of Pathology, Children's Hospital of Nanjing Medical University, Nanjing, China; ^2^Department of Clinical Laboratory, Molecular Epidemiology Laboratory, Harbin Medical University Cancer Hospital, Harbin, China; ^3^Department of Pathology, Anhui Provincial Children's Hospital, Hefei, China; ^4^Department of Hematology, The Second Affiliated Hospital and Yuying Children's Hospital of Wenzhou Medical University, Wenzhou, China; ^5^Department of Pediatric Surgery, Guangzhou Institute of Pediatrics, Guangzhou Women and Children's Medical Center, Guangzhou Medical University, Guangzhou, China

**Keywords:** *LMO1*, polymorphism, neuroblastoma, susceptibility, eastern Chinese Han

## Abstract

**Background:** Neuroblastoma, a neuroendocrine tumor, stems from the developing sympathetic nervous system. Previous genome-wide association studies (GWASs) have discovered a number of neuroblastoma susceptibility genes in Caucasians including *LIM domain only 1 (LMO1)*.

**Objective:** We conducted a three-center case-control study including 313 cases and 716 controls with the purpose to evaluate the association between five GWAS-identified *LMO1* variants (rs110419 A>G, rs4758051 G>A, rs10840002 A>G, rs204938 A>G, and rs2168101 G>T) and neuroblastoma susceptibility in eastern Chinese children.

**Methods:** Odds ratios (ORs) and 95% confidence intervals (CIs) were calculated to evaluate the strength of the associations. False positive report possibility (FPRP) analysis was performed to check whether significant results were noteworthy.

**Results:** Significant associations with neuroblastoma risk were found for four (rs110419, rs4758051, rs10840002, and rs2168101) out of the five polymorphisms. Combined analysis demonstrated that carriers of 4–5 protective genotypes had a significantly decreased risk of neuroblastoma in comparison those with 0–3 protective genotypes (adjusted OR = 0.51, 95% CI = 0.39–0.68, *P* < 0.0001). Haplotype analysis of the five SNPs yield four significant haplotypes associated with neuroblastoma susceptibility.

**Conclusion:** In conclusion, we confirmed *LMO1* polymorphisms may reduce neuroblastoma risk in eastern Chinese populations.

## Introduction

Neuroblastoma is the most frequently diagnosed solid tumor outside of cranium in childhood, especially in the first year of life (1–3). A median age at diagnosis of this disease is about 17 months. Neuroblastoma is a type of neuroendocrine tumor, originating from the developing sympathetic nervous system. Its incidence rate varies worldwide, affecting ~8–14 individuals per million in Western countries (4) and 7.7 per million in China (5). Neuroblastoma is a group of heterogeneous diseases. Majority of tumors (65%) occur in the abdomen, followed by the neck, pelvis, and chest (2). The clinic outcomes are age-dependent, varying greatly from spontaneous regression of tumor in infants to unfavorable prognosis in older children even after intensive multimodality treatments. Neuroblastoma patients are traditionally categorized into low-, intermediate-, and high-risk groups. To more accurately group patients, the International Neuroblastoma Risk Group (INRG) classification has yield 16 risk groups, by integrating clinical and molecular characteristics, such as age at diagnosis, tumor stage, histopathology, as well as genetic signatures (6). Environmental factors for neuroblastoma are not well-established. Environmental exposures, such as exposures to drugs and hair dyes during maternity and pregnancy, may increase the risk of the disease, to a small extent (7).

Neuroblastoma is genetically heterogeneous (8). Lately, intensive research has led to dramatic discoveries of some causal heritable mutations in familial neuroblastoma (9). These hallmark advances include mutations in the anaplastic lymphoma kinase (*ALK*) receptor tyrosine kinase gene and inactivating mutations in *Paired-like homeobox 2b* (*PHOX2B*), a transcription factor essentially regulating neural crest development. However, it should be noted that familial subset only constitutes up to 10% of neuroblastoma cases. More studies are needed to further define the genetic basis of neuroblastoma, particularly for sporadic cases. Previous genome-wide association studies (GWASs) have discovered 9 neuroblastoma susceptibility genes in Caucasians, including *CASC15, BARD1, LIN28B, LMO1, DUSP12, IL31RA, DDX4, HSD17B12*, and *HACE1* (10–12).

*LIM domain only 1* (*LMO1*) at 11p15.4 belongs to *LMO* gene family, all members of which (*LMO1*-*4*) are involved in carcinogenesis. The name of LIM domain originated from the LIN-11, ISL-1, and MEC-3 proteins in *Caenorhabditis elegans* (13, 14). *LMO* encodes a cysteine-rich transcriptional regulator composed of two zinc finger LIM domains but little other sequence (10, 15). Gene amplification (duplication) was observed in 12.4% of 701 neuroblastoma patients with primary tumor (10). A number of neuroblastoma susceptibility loci have been identified in this gene by GWAS. *LMO1* rs110419 A>G, rs4758051 G>A, rs10840002 A>G, and rs204938 A>G were identified in a GWAS with 2,251 neuroblastoma patients and 6,097 controls of European ancestry (10). The rs2168101 G>T located in the *LMO1* super-enhancer was also reported to modify neuroblastoma susceptibility by the same research group (15). In order to investigate the association between these *LMO1* polymorphisms and neuroblastoma in eastern Chinese children, we performed a multi-center study with 313 cases and 716 controls recruited from three regions of East China.

## Methods and materials

### Study population

We carried out a multi-center case control study with 313 cases and 762 controls by incorporating participants from Jiangsu (158 cases and 426 controls, Supplemental Table 1) and Anhui (119 cases and 264 controls, Supplemental Table 2) provinces, as well as Wenzhou area (36 cases and 72 controls, Supplemental Table 3) (16, 17). The detailed descriptions of participants were elaborated in previous studies. All cases had primary tumors that were histologically confirmed to be neuroblastoma. Age and sex-matched controls were separately collected in each participating center. Each participant or his/her guardian was obligated to sign informed consent before blood sample collection. Approvals of the study protocols were issued by individual Institutional Review Board of participating centers.

### Polymorphism selection and genotyping

SNPs in the *LMO1* gene were selected by literature review. In total, five SNPs in the *LMO1* gene (rs110419, rs4758051, rs10840002, rs204938, rs2168101) were analyzed. The first four SNPs were identified in a GWAS with 2,251 neuroblastoma patients and 6,097 controls of European ancestry (10). The last one is a SNP located in the *LMO1* super-enhancer, which was also reported to modify neuroblastoma susceptibility by the same research group (15).

Genomic DNAs were extracted from blood samples, quantified, and following standardized procedures (18–21). TaqMan assay was performed for SNP genotyping with allele-specific probes tagged with the fluorescent dyes VIC and FAM, respectively (22–24). All assays were run in 384-well plates on the ABI 7900 HT Sequence Detection System (Applied Biosystems, Foster City, CA). Quality control was applied as reported before (25–27).

### Statistical analysis

Departure from Hardy-Weinberg equilibrium (HWE) was examined for each SNP using a good-of-fit χ^2^ in control subjects. The two-sided χ^2^ test was used to compare the differences in the categorical variables between cases and controls, including age, gender, sites of origin, and genotype frequency. The strength of association of tested SNPs and neuroblastoma was assessed by conducting logistic regression analysis to calculate odds ratios (ORs) and 95% confidence intervals (CIs). We also carried out the false positive report probability (FPRP) analysis to check whether significant results are noteworthy as described previously (28). Briefly, three parameters were employed to determine FPRP values, prior probability π representing a true association between the SNP and a disease, statistical power, and *P*-value (29). A FPRP threshold of 0.2 was preset for this study. We chose *P* < 0.05 as a standard of statistical significance. All *P*-values were two-sided. Statistics were completed with STATA (version 11.0; Stata Corporation, College Station, TX).

## Results

### Population characters

A total of 313 cases and 762 controls were recruited for this association study (Table 1). Prior to study, case and controls were subjected to frequency matching to ensure there was no significant difference in the age (*P* = 0.823) and gender (*P* = 0.610) between cases and controls. Neuroblastoma may stem from different sites of the body. Based on sites of origin, cases were divided into adrenal gland (68, 21.73%), retroperitoneal region (126, 40.26%), mediastinum (99, 31.63%), and other region (20, 6.39%) groups.

**Table 1:** Demographic characteristics of neuroblastoma patients and cancer-free controls for eastern Chinese children.

**Variables**	**Cases (*****n*** = **313)**		**Controls (*****n*** = **762)**		***P^*a*^***
	**No**.	**%**	**No**.	**%**	
Age range, month	0.001–132.00		0.001–132.00		0.823
Mean ± SD	29.72 ± 28.21		33.04 ± 30.30		
≤ 18	142	45.37	340	44.62	
>18	171	54.63	422	55.38	
Gender					0.610
Female	145	46.33	340	44.62	
Male	168	53.67	422	55.38	
**SITES OF ORIGIN**					
Adrenal gland	68	21.73			
Retroperitoneal region	126	40.26			
Mediastinum	99	31.63			
Other region	20	6.39			

a *Two-sided χ^2^ test between neuroblastoma patients and cancer-free controls*.

### Association between *LMO1* polymorphisms and neuroblastoma susceptibility

No deviation from HWE was found for any studied SNPs (*P* = 0.405 for rs110419, *P* = 0.530 for rs4758051, *P* = 0.981 for rs10840002, *P* = 0.336 for rs204938, *P* = 0.389 for rs2168101). Single locus analysis indicated that four out of the five polymorphisms were significantly associated with neuroblastoma risk (Table 2). First, *LMO1* rs110419 was shown to significantly decrease the risk of neuroblastoma (heterogeneous: adjusted OR = 0.62, 95% CI = 0.46–0.82; homogenous: adjusted OR = 0.65, 95% CI = 0.44–0.97; additive: adjusted OR = 0.76, 95% CI = 0.63–0.92; dominant: adjusted OR = 0.63, 95% CI = 0.48–0.82). Second, a protective association with neuroblastoma susceptibility was also found for rs4758051 (heterogeneous: adjusted OR = 0.62, 95% CI = 0.47–0.84; homogenous: adjusted OR = 0.67, 95% CI = 0.46–0.99; additive: adjusted OR = 0.78, 95% CI = 0.64–0.94; dominant: adjusted OR = 0.64, 95% CI = 0.49–0.84). Third, the rs10840002 conferred decreased susceptibility to neuroblastoma (heterogeneous: adjusted OR = 0.68, 95% CI = 0.50–0.91; dominant: adjusted OR = 0.74, 95% CI = 0.56–0.97). Finally, we found that rs2168101 was significantly associated with a decreased neuroblastoma risk (heterogeneous: adjusted OR = 0.50, 95% CI = 0.38–0.67; homogenous: adjusted OR = 0.52, 95% CI = 0.28–0.96; additive: adjusted OR = 0.59, 95% CI = 0.46–0.74; dominant: adjusted OR = 0.51, 95% CI = 0.38–0.67). Genotypes with variant alleles were referred to as protective genotypes. While the five SNPs were combined, we found that carriers of 4–5 protective genotypes had a significantly decreased risk of neuroblastoma at an OR of 0.51 (95% CI = 0.39–0.68).

**Table 2:** *LMO1* gene polymorphisms and neuroblastoma susceptibility in eastern Chinese population.

**Genotype**	**Cases (*n* = 313)**	**Controls (*n* = 762)**	***P*^a^**	**Crude OR (95% CI)**	***P***	**Adjusted OR (95% CI)^b^**	***P*^b^**
**RS110419 (HWE** = **0.405)**							
AA	150 (47.92)	279 (36.61)		1.00		1.00	
AG	118 (37.70)	355 (46.59)		**0.62 (0.46–0.82)**	**0.0011**	**0.62 (0.46–0.82)**	**0.0010**
GG	45 (14.38)	128 (16.80)		**0.65 (0.44–0.97)**	**0.034**	**0.65 (0.44–0.97)**	**0.035**
Additive			0.0026	**0.76 (0.63–0.92)**	**0.0041**	**0.76 (0.63–0.92)**	**0.0040**
Dominant	163 (52.08)	483 (63.39)	0.0006	**0.63 (0.48–0.82)**	**0.0006**	**0.63 (0.48–0.82)**	**0.0006**
Recessive	268 (85.62)	634 (83.20)	0.326	0.83 (0.58–1.20)	0.327	0.83 (0.58–1.21)	0.334
**RS4758051 (HWE** = **0.530)**							
GG	138 (44.09)	256 (33.60)		1.00		1.00	
AG	123 (39.30)	364 (47.77)		**0.63 (0.47–0.84)**	**0.0017**	**0.62 (0.47–0.84)**	**0.0015**
AA	52 (16.61)	142 (18.64)		**0.68 (0.47–0.99)**	**0.046**	**0.67 (0.46–0.99)**	**0.042**
Additive			0.0048	**0.78 (0.65–0.94)**	**0.0094**	**0.78 (0.64–0.94)**	**0.0085**
Dominant	175 (55.91)	506 (66.40)	0.0012	**0.64 (0.49–0.84)**	**0.0012**	**0.64 (0.49–0.84)**	**0.0011**
Recessive	261 (83.39)	620 (81.36)	0.434	0.87 (0.61–1.23)	0.434	0.87 (0.61–1.23)	0.417
**RS10840002 (HWE** = **0.981)**							
AA	120 (38.34)	240 (31.50)		1.00		1.00	
AG	128 (40.89)	375 (49.21)		**0.68 (0.51–0.92)**	**0.012**	**0.68 (0.50–0.91)**	**0.011**
GG	65 (20.77)	147 (19.29)		0.88 (0.61–1.27)	0.509	0.88 (0.61–1.27)	0.491
Additive			0.036	0.90 (0.74–1.08)	0.264	0.90 (0.75–1.08)	0.251
Dominant	193 (61.66)	522 (68.50)	0.031	**0.74 (0.56–0.97)**	**0.031**	**0.74 (0.56–0.97)**	**0.028**
Recessive	248 (79.23)	615 (80.71)	0.581	1.10 (0.79–1.52)	0.581	1.10 (0.79–1.52)	0.589
**RS204938 (HWE** = **0.336)**							
AA	200 (63.90)	476 (62.47)		1.00		1.00	
AG	97 (30.99)	258 (33.86)		0.90 (0.67–1.19)	0.446	0.91 (0.68–1.21)	0.498
GG	16 (5.11)	28 (3.67)		1.36 (0.72–2.57)	0.343	1.34 (0.71–2.54)	0.368
Additive			0.418	1.00 (0.79–1.26)	1.000	1.01 (0.80–1.27)	0.969
Dominant	113 (36.10)	286 (37.53)	0.659	0.94 (0.72–1.24)	0.659	0.95 (0.72–1.25)	0.709
Recessive	297 (94.89)	734 (96.33)	0.280	1.41 (0.75–2.65)	0.282	1.39 (0.74–2.61)	0.311
**RS2168101 (HWE** = **0.389)**							
GG	214 (68.37)	401 (52.62)		1.00		1.00	
GT	85 (27.16)	310 (40.68)		**0.51 (0.38–0.69)**	<**0.0001**	**0.50 (0.38–0.67)**	<**0.0001**
TT	14 (4.47)	51 (6.69)		**0.51 (0.28–0.95)**	**0.034**	**0.52 (0.28–0.96)**	**0.036**
Additive			< 0.0001	**0.59 (0.47–0.75)**	<**0.0001**	**0.59 (0.46–0.74)**	<**0.0001**
Dominant	99 (31.63)	361 (47.38)	< 0.0001	**0.51 (0.39–0.68)**	<**0.0001**	**0.51 (0.38–0.67)**	<**0.0001**
Recessive	299 (95.53)	711 (93.31)	0.165	0.65 (0.36–1.20)	0.169	0.66 (0.36–1.21)	0.182
**COMBINED EFFECT OF PROTECTIVE GENOTYPES**^C^							
0–3	213 (68.05)	401 (52.62)		1.00		1.00	
4–5	100 (31.95)	361 (47.38)	< 0.0001	**0.52 (0.40–0.69)**	< 0.0001	**0.51 (0.39–0.68)**	< 0.0001

a *χ^2^ test for genotype distributions between neuroblastoma patients and controls*.

b *Adjusted for age and gender*.

c *Protective genotypes were rs110419 AG/GG, rs4758051 AG/AA, rs10840002 AG/GG, rs204938 AG/GG, and rs2168101 GT/TT*.

*The results were in bold if the 95% CI excluded 1 or P < 0.05*.

### Stratified analysis

Significant SNPs were further subjected to stratified analysis by age, gender, and sites of origin (Table 3). Protective association between SNPs and neuroblastoma risk were observed in all age- and gender-groups for rs110419, rs2168101, and combined analysis, but only in children >18 and males for rs4758051. In term of sites of origin, all SNPs and combination were shown to decrease the risk of develop tumor in retroperitoneal region. Additionally, children carrying rs2168101 or 4–5 protective genotypes were also less likely develop tumor in mediastinum. For rs10840002, significant result only remained in the group of retroperitoneal region.

**Table 3:** Stratification analysis for the association between protective genotypes and neuroblastoma susceptibility.

**Variables**	**rs110419**		**rs4758051**		**rs10840002**		**rs2168101**		**Protective genotypes**	
	**AG/GG vs. AA**		**AG/AA vs. GG**		**AG/GG vs. AA**		**GT/TT vs. GG**		**4–5 vs. 0–3**	
	**AOR (95% CI) ^a^**	***P*^a^**	**AOR (95% CI) ^a^**	***P*^a^**	**AOR (95% CI) ^a^**	***P*^a^**	**AOR (95% CI) ^a^**	***P*^a^**	**AOR (95% CI) ^a^**	***P*^a^**
**AGE, MONTH**										
≤ 18	**0.62 (0.42–0.93)**	**0.020**	0.71 (0.47–1.07)	0.099	0.73 (0.48–1.12)	0.147	**0.65 (0.44–0.97)**	**0.034**	**0.60 (0.40–0.90)**	**0.013**
>18	**0.63 (0.44–0.90)**	**0.011**	**0.58 (0.41–0.84)**	**0.003**	0.73 (0.51–1.05)	0.089	**0.40 (0.27–0.60)**	<**0.0001**	**0.45 (0.30–0.66)**	<**0.0001**
**GENDER**										
Females	**0.64 (0.43–0.96)**	**0.030**	0.67 (0.45–1.01)	0.054	0.69 (0.46–1.04)	0.073	**0.60 (0.40–0.90)**	**0.013**	**0.58 (0.39–0.88)**	**0.009**
Males	**0.62 (0.43–0.89)**	**0.009**	**0.60 (0.41–0.86)**	**0.006**	0.75 (0.51–1.09)	0.129	**0.43 (0.29–0.64)**	<**0.0001**	**0.46 (0.32–0.68)**	<**0.0001**
**SITES OF ORIGIN**										
Adrenal gland	0.99 (0.59–1.67)	0.983	0.71 (0.43–1.18)	0.192	0.73 (0.44–1.23)	0.236	0.60 (0.36–1.00)	0.052	0.72 (0.44–1.20)	0.209
Retroperitoneal	**0.46 (0.31–0.67)**	<**0.0001**	**0.49 (0.34–0.72)**	**0.0003**	**0.61 (0.42–0.90)**	**0.013**	**0.47 (0.32–0.71)**	**0.0003**	**0.41 (0.27–0.62)**	<**0.0001**
Mediastinum	0.67 (0.44–1.01)	0.058	0.89 (0.57–1.37)	0.585	1.01 (0.64–1.58)	0.983	**0.50 (0.32–0.79)**	**0.0027**	**0.55 (0.36–0.86)**	**0.009**
Others	0.71 (0.29–1.73)	0.444	0.50 (0.20–1.21)	0.123	0.55 (0.22–1.34)	0.185	0.47 (0.18–1.23)	0.124	0.46 (0.18–1.22)	0.120

a *Adjusted for age and gender*.

*The results were in bold if the 95% CI excluded 1 or P < 0.05*.

### FPRP analysis

Results of association between genetic variants and diseases may be subjected to false positivity. Here, we performed FPRP analysis to interrogate our significant findings (Table 4). An FPRP noteworthiness value below 0.2 was prespecified for each association (29). When prior probability of 0.1 was adopted, significant association for rs110419 A>G (AG/GG vs. AA) remained noteworthy, so were results for retroperitoneal subgroup. All results for rs4758051 G>A were deserving of attention, with an exception of homogeneous model (AA vs. GG). Noteworthy results were also found for the rs10840002 A>G (AG vs. AA) and the rs2168101 G>T except for homogeneous model (TT vs. GG) and females. In the combined analysis, findings for 4–5 vs. 0–3 protective genotypes, children>18, males and retroperitoneal subgroup could be called noteworthy.

**Table 4:** False-positive report probability analysis for the significant findings.

**Genotype**	**Crude OR (95% CI)**	***P*^a^**	**Statistical power^b^**	**Prior probability**				
				**0.25**	**0.1**	**0.01**	**0.001**	**0.0001**
**RS110419 A**>**G**								
AG vs. AA	0.62 (0.46–0.82)	0.001	0.361	**0.009**	0.027	0.232	0.753	0.968
GG vs. AA	0.65 (0.44–0.97)	0.034	0.458	**0.184**	0.403	0.881	0.987	0.999
AG/GG vs. AA	0.63 (0.48–0.82)	0.0006	0.317	**0.006**	**0.017**	**0.158**	0.654	0.950
≤ 18	0.63 (0.42–0.94)	0.022	0.377	**0.148**	0.343	0.852	0.983	0.998
>18	0.63 (0.44–0.90)	0.011	0.358	**0.081**	0.209	0.744	0.967	0.997
Females	0.64 (0.43–0.95)	0.025	0.400	**0.158**	0.361	0.861	0.984	0.998
Males	0.62 (0.43–0.89)	0.009	0.329	**0.073**	0.190	0.721	0.963	0.996
Retroperitoneal	0.46 (0.32–0.68)	< 0.0001	0.031	**0.007**	**0.020**	**0.181**	0.690	0.957
**RS4758051 G**>**A**								
AG vs. GG	0.63 (0.47–0.84)	0.002	0.415	**0.012**	**0.036**	0.289	0.804	0.976
AA vs. GG	0.68 (0.47–0.99)	0.046	0.547	**0.200**	0.429	0.892	0.988	0.999
AA vs. GG/AG	0.64 (0.49–0.84)	0.001	0.377	**0.009**	**0.028**	0.240	0.761	0.970
>18	0.59 (0.41–0.84)	0.004	0.236	**0.045**	**0.123**	0.608	0.940	0.994
Males	0.60 (0.41–0.86)	0.006	0.271	**0.061**	**0.164**	0.683	0.956	0.995
Retroperitoneal	0.49 (0.34–0.72)	0.0002	0.054	**0.011**	**0.033**	0.270	0.789	0.974
**RS10840002 A**>**G**								
AG vs. AA	0.68 (0.51–0.92)	0.012	0.666	**0.050**	**0.138**	0.637	0.947	0.994
GG/AG vs. AA	0.74 (0.56–0.97)	0.031	0.764	**0.109**	0.268	0.801	0.976	0.998
Retroperitoneal	0.61 (0.42–0.90)	0.013	0.330	**0.103**	0.256	0.791	0.974	0.997
**RS2168101 G**>**T**								
GT vs. GG	0.51 (0.38–0.69)	< 0.0001	0.045	**0.001**	**0.002**	**0.017**	**0.146**	0.631
TT vs. GG	0.51 (0.28–0.95)	0.034	0.192	0.346	0.614	0.946	0.994	0.999
GT/TT vs. GG	0.51 (0.39–0.68)	< 0.0001	0.035	**0.000**	**0.001**	**0.007**	**0.067**	0.419
≤ 18	0.66 (0.44–0.98)	0.040	0.470	**0.203**	**0.434**	0.894	0.988	0.999
>18	0.40 (0.27–0.60)	< 0.0001	0.008	**0.002**	**0.007**	**0.074**	0.445	0.889
Females	0.62 (0.42–0.93)	0.020	0.363	**0.143**	0.333	0.846	0.982	0.998
Males	0.43 (0.29–0.64)	< 0.0001	0.016	**0.004**	**0.011**	**0.113**	0.563	0.928
Retroperitoneal	0.48 (0.32–0.72)	0.0004	0.062	**0.019**	**0.055**	0.392	0.867	0.985
Mediastinum	0.51 (0.32–0.79)	0.003	0.120	**0.068**	**0.179**	0.706	0.960	0.996
**PROTECTIVE GENOTYPES**								
4–5 vs. 0–3	0.52 (0.40–0.69)	< 0.0001	0.042	**0.000**	**0.001**	**0.009**	**0.084**	0.479
≤ 18	0.61 (0.41–0.91)	0.014	0.320	**0.118**	0.287	0.816	0.978	0.998
>18	0.45 (0.31–0.66)	< 0.0001	0.027	**0.006**	**0.018**	**0.171**	0.675	0.954
Females	0.60 (0.40–0.90)	0.013	0.300	**0.113**	0.277	0.809	0.977	0.998
Males	0.46 (0.32–0.68)	< 0.0001	0.033	**0.007**	**0.020**	**0.181**	0.690	0.957
Retroperitoneal	0.41 (0.27–0.62)	< 0.0001	0.014	**0.006**	**0.019**	**0.173**	0.678	0.955
Mediastinum	0.56 (0.36–0.86)	0.009	0.211	**0.113**	0.277	0.808	0.977	0.998

a *Chi-square test was used to calculate the genotype frequency distributions*.

b *Statistical power was calculated using the number of observations in the subgroup and the OR and P-values in this table*.

*The results were in bold if the 95% CI excluded 1 or P < 0.05*.

### *LMO1* haplotypes and neuroblastoma risk

We next further explored whether the haplotypes of the five *LMO1* SNPs would modify neuroblastoma risk. As presented in Table 5, 19 *LMO1* haplotypes were available for analysis. When compared to reference haplotype AGAAG, four haplotypes, AGGAG, GGGAG, GAGAT, and GAGGT were significantly associated with altered neuroblastoma risk.

**Table 5:** Association between *LMO1* haplotypes and neuroblastoma susceptibility.

**Haplotype^a^**	**Case (*n* = 626)**	**Control (*n* = 1,524)**	**Crude OR (95% CI)**	***P***	**Adjusted OR^b^ (95% CI)**	***P*^b^**
	**No. (%)**	**No. (%)**				
AGAAG	271 (43.29)	606 (39.76)	1.00		1.00	
AGAAT	0 (0.00)	2 (0.13)	/	/	/	/
AGAGG	33 (5.27)	67 (4.40)	1.10 (0.71–1.71)	0.668	1.10 (0.71–1.72)	0.664
AGGAG	16 (2.56)	10 (0.66)	**3.58 (1.60–7.99)**	**0.002**	**3.59 (1.60–8.05)**	**0.002**
AGGGG	7 (1.12)	8 (0.52)	1.96 (0.70–5.45)	0.199	2.03 (0.73–5.68)	0.176
AGGGT	0 (0.00)	1 (0.07)	/	/	/	/
AAAAG	1 (0.16)	5 (0.33)	0.45 (0.05–3.85)	0.464	0.46 (0.05–3.99)	0.483
AAGAG	54 (8.63)	156 (10.24)	0.77 (0.55–1.09)	0.141	0.77 (0.55–1.08)	0.131
AAGAT	0 (0.00)	1 (0.07)	/	/	/	/
AAGGG	36 (5.75)	57 (3.74)	1.41 (0.91–2.20)	0.125	1.43 (0.92–2.22)	0.115
GGAAG	39 (6.23)	106 (6.96)	0.82 (0.56–1.22)	0.332	0.83 (0.56–1.22)	0.339
GGAAT	9 (1.44)	24 (1.57)	0.84 (0.39–1.83)	0.658	0.83 (0.38–1.81)	0.633
GGAGG	12 (1.92)	37 (2.43)	0.73 (0.37–1.41)	0.345	0.73 (0.38–1.43)	0.359
GGAGT	0 (0.00)	6 (0.39)	/	/	/	/
GGGAG	6 (0.96)	1 (0.07)	**13.41 (1.61–111.99)**	**0.017**	**15.04 (1.79–126.19)**	**0.013**
GGGAT	3 (0.48)	5 (0.33)	1.34 (0.32–5.65)	0.689	1.25 (0.30–5.29)	0.762
GGGGG	2 (0.32)	1 (0.07)	4.47 (0.40–49.53)	0.222	4.56 (0.41–50.74)	0.217
GGGGT	1 (0.16)	2 (0.13)	1.12 (0.10–12.38)	0.928	1.14 (0.10–12.71)	0.914
GAAAG	2 (0.32)	1 (0.07)	4.47 (0.40–49.53)	0.222	4.47 (0.40–49.62)	0.223
GAAAT	1 (0.16)	1 (0.07)	2.24 (0.14–35.88)	0.570	2.60 (0.16–40.19)	0.518
GAGAG	21 (3.35)	29 (1.90)	1.62 (0.91–2.89)	0.103	1.66 (0.93–2.97)	0.087
GAGAT	74 (11.82)	263 (17.26)	**0.63 (0.47–0.85)**	**0.002**	**0.63 (0.47–0.84)**	**0.002**
GAGGG	13 (2.08)	28 (1.84)	1.04 (0.53–2.04)	0.913	1.05 (0.53–2.06)	0.895
GAGGT	25 (3.99)	107 (7.02)	**0.52 (0.33–0.83)**	**0.006**	**0.52 (0.33–0.82)**	**0.005**

a *The haplotype order were rs110419 A>G, rs4758051 G>A, rs10840002 A>G, rs204938 A>G, and rs2168101 G>T*.

b *Adjusted for age and gender*.

*The results were in bold if the 95% CI excluded 1 or P < 0.05*.

## Discussion

The replication study is the golden method for the validation of the genetic associations. Therefore, we performed this multi-center case control study to examine the role of five GWAS-identified neuroblastoma susceptibility polymorphisms in Chinese children recruited from East China. We confirmed that four variants (rs110419, rs4758051, rs10840002, and rs2168101) in the *LMO1* gene were associated with a decreased risk of neuroblastoma in our study population.

Functions of LMO family member remain to be clarified. These proteins consist of two LIM domains that can interact with a variety of proteins to regulate cancer-related cellular events including cell cycle, self-renewal, and metastasis (14). LMO1–4 are engaged in a broad spectrum of developmental processes, and found to be involved in the initiation or the progression of T cell leukemia, breast cancer as well as neuroblastoma, to date (30, 31). LIM domain is characterized as a form of zinc finger mediating protein-protein interacting, but not binding DNA sequences (13, 14). They may modulate transcriptional processes by nucleating and regulating transcription factor complexes (14).

*LMO1* at 11p15.4 was identified as a neuroblastoma susceptibility locus in a previous GWAS (10). The association between *LMO1* SNPs with neuroblastoma susceptibility was replicated in several independent cohorts from US, UK, and Italy (10). Clinical analysis indicated that the risk alleles of *LMO1* (rs110419 A, rs4758051 G, rs10840002 A, rs204938 C) were in significant association with metastatic and high-risk neuroblastoma. Genotype and phenotype correlation analysis revealed that rs110419 A (risk allele) was significantly associated with elevated *LMO1* expression. *LMO1 s*omatic copy number gain also led to increased mRNA and protein expression accordingly. *In vitro* study further showed that loss- or gain-of-function of *LMO1* suppressed and promoted proliferation of neuroblastoma cell lines, respectively (10). The rs2168101 polymorphism is positioned in a super-enhancer of *LMO1*. The G allele is a risk allele that constitutes a conserved GATA box to facilitate transcription factor binding (e.g., GATA3). The variant A allele eradicates GATA3 binding site and associates with a decrease in the *LMO1* expression levels in neuroblastoma primary tumor. These biological findings are consistent with the protective association of with neuroblastoma susceptibility (15).

A recent study reported that transgenic expression of LMO1 alone failed to induce tumorigenesis in a zebra fish model, but acted as a synergist of MYCN, an oncogene amplified in ~20% of neuroblastoma (32). Eighty percent of transgenic fish with overexpression of both LMO1 and MYCN developed tumors by 24 weeks of age in comparison with 20–30% of the fish expressing MYCN alone. LMO1 cooperated with MYCN to induce abnormal proliferation of sympathoadrenal cells in the inter-renal gland and enhance metastasis. Moreover, LMO1 was found to confer human neuroblastoma cells invasive and migratory attributes. RNA sequencing (RNA-seq) and gene set enrichment analysis (GSEA) indicated that matrisome, extracellular matrix-associated proteins, integrins coding signature genes were enriched in the neuroblastoma cell lines expression high levels of LMO1. Later on, a mechanistic study using chromatin immunoprecipiation and DNA sequencing demonstrated that in neuroblastoma, LMO1 could affect *LIMSI* (*LIM and senescent cell antigen-like domains 1*), *Ras suppressor protein 1* (*RSU1*), and *relaxin 2* (*RLN2*) gene, and regulate a carcinogenic LIMSI/integin-linked kinase pathway (33).

Since *LMO1* was established as a neuroblastoma risk gene, the association between *LMO1* SNPs and neuroblastoma susceptibility has been replicated in several different ethnic groups (34–39). The first four *LMO1* SNPs were tested for association in 390 African-American neuroblastoma patients and 2,500 controls; however, no significant association was observed (34). Moreover, the two most significant GWAS-identified SNPs in *LMO1* (intronic rs110419 and intergenic rs4758051) were further replication in 370 cases and 809 controls from Italy and significant association was detected for rs110419 (35). Lu et al examine 11 *LMO1* SNPs including rs110419 and rs204938 in a study population of 244 neuroblastoma patients and 305 healthy controls from North China but no significant association were found for these two SNPs (36). *LMO1* rs110419 A>G, rs4758051 G>A, rs10840002 A>G, and rs204938 A>G) were analyzed in 256 neuroblastoma cases and 531 controls accrued from South China. Only rs110419 was associated with a decreased neuroblastoma risk (37). Replication study with 118 cases and 281 controls from North China reported negative association of neuroblastoma risk with rs4758051 and rs10840002. Moreover, the association between the *LMO1* super-enhancer polymorphism rs2168101 and a decreased neuroblastoma risk was validated in the southern and northern Chinese population alone as well as in the combined case series (39).

As shown in literature review and our results above, association results between the same variant and disease varies among different ethnicities, even among different China Han groups from different geographical regions. Several reasons may affect association results and cause discrepancies, including relative modest effects of the SNPs, limited sample sizes of case control studies, different genetic backgrounds among different ethnicities, as well as different environmental exposure and life styles among different geographical regions. Therefore, our findings should be explained cautiously, and cannot be extrapolated to other populations prior to validation studies.

Limitations of this study should be discussed. First, although we included samples from three independent medical centers, sample size in this study is still relative small due to low incidence of this disease. Second, due to the retrospective nature of the study, many precious environmental factors that participants exposed to were not available for further analysis. Finally, functional analysis should be performed to investigate underlying biological mechanisms of *LMO1* SNPs' protective effects on neuroblastoma.

In conclusion, we found that four *LMO1* SNPs were associated with a decreased neuroblastoma risk in eastern China populations. Our findings warrant further validation in large well-designed studies.

## Author contributions

JH and HW conceived and designed the study. FH, YT, CZ, JD, YW, and HZ collected the samples and information. LH, JZ, and JH performed the experiment, analyzed the data and prepared the tables, and wrote the manuscript. All authors reviewed the manuscript. In addition, all authors have read and approved the manuscript.

### Conflict of interest statement

The authors declare that the research was conducted in the absence of any commercial or financial relationships that could be construed as a potential conflict of interest.
