# Supplementary material for: LMO1 Gene Polymorphisms Reduce Neuroblastoma Risk in Eastern Chinese Children: A Three-Center Case-Control Study
Source: Front Oncol. 2018 Oct 23;8:468. doi: 10.3389/fonc.2018.00468 (PMC6206234; doi:10.3389/fonc.2018.00468)
Supplement: Supplementary file 1 [file Table_1.DOC]

| **Supplemental Table 1**. Demographic characteristics of neuroblastoma patients and cancer-free controls from Jiangsu province | | | | | |
| --- | --- | --- | --- | --- | --- |
| Variables | Cases (n=158) | | Controls (n=426) | | *P ^a^* |
|  | No. | % | No. | % |  |
| Age range, month | 0.01-132.00 | | 0.17-132.00 | | 0.656 |
| Mean ± SD | 34.03 ± 32.19 | | 36.73 ± 33.16 | |  |
| ≤18 | 70 | 44.30 | 180 | 42.25 |  |
| >18 | 88 | 55.70 | 246 | 57.75 |  |
| Gender |  |  |  |  | 0.410 |
| Female | 78 | 49.37 | 194 | 45.54 |  |
| Male | 80 | 50.63 | 232 | 54.46 |  |
| Sites of origin |  |  |  |  |  |
| Adrenal gland | 14 | 8.86 |  |  |  |
| Retroperitoneal region | 76 | 48.10 |  |  |  |
| Mediastinum | 59 | 37.34 |  |  |  |
| Other region | 9 | 5.70 |  |  |  |
| SD, standard deviation.  ^a^ Two-sided *χ^2^* test between neuroblastoma patients and cancer-free controls. | | | | | |

| **Supplemental Table 2**. Demographic characteristics for neuroblastoma patients and cancer-free controls from Anhui province | | | | | |
| --- | --- | --- | --- | --- | --- |
| Variables | Cases (N=119) | | Controls (N=264) | | *P^a^* |
|  | No. | % | No. | % |  |
| Age range, month | 0.001-108 | | 0.001-96 | | 0.507 |
| Mean ± SD | 26.86 ± 23.19 | | 29.67 ± 27.52 | |  |
| ≤18 | 52 | 43.70 | 125 | 47.35 |  |
| >18 | 67 | 56.30 | 139 | 52.65 |  |
| Gender |  |  |  |  | 0.941 |
| Female | 50 | 42.02 | 112 | 42.42 |  |
| Male | 69 | 57.98 | 152 | 57.58 |  |
| Clinical stages |  |  |  |  |  |
| I | 45 | 37.82 |  |  |  |
| II | 52 | 43.70 |  |  |  |
| III | 14 | 11.76 |  |  |  |
| IV | 8 | 6.72 |  |  |  |
| Sites of origin |  |  |  |  |  |
| Adrenal gland | 43 | 36.13 |  |  |  |
| Retroperitoneal region | 41 | 34.45 |  |  |  |
| Mediastinum | 26 | 21.85 |  |  |  |
| Other region | 9 | 7.56 |  |  |  |
| SD, standard deviation.  ^a^ Two-sided *χ^2^* test for distributions between neuroblastoma patients and controls. | | | | | |

| **Supplemental Table 3**. Demographic characteristics for neuroblastoma patients and cancer-free controls from Wenzhou area | | | | | |
| --- | --- | --- | --- | --- | --- |
| Variables | Cases (n=36) | | Controls (n=72) | | *P ^a^* |
|  | No. | % | No. | % |  |
| Age range, month | 0.05-72 | | 8-72 | | 0.496 |
| Mean ± SD | 20.25 ± 20.73 | | 23.58 ± 15.36 | |  |
| ≤18 | 20 | 55.56 | 35 | 48.61 |  |
| >18 | 16 | 44.44 | 37 | 51.39 |  |
| Gender |  |  |  |  | 1.000 |
| Female | 17 | 47.22 | 34 | 47.22 |  |
| Male | 19 | 52.78 | 38 | 52.78 |  |
| Clinical stages |  |  |  |  |  |
| I | 15 | 41.67 | / | / |  |
| II | 2 | 5.56 | / | / |  |
| III | 9 | 25.00 | / | / |  |
| IV | 7 | 19.44 | / | / |  |
| 4s | 3 | 8.33 | / | / |  |
| Sites of origin |  |  |  |  |  |
| Adrenal gland | 11 | 30.56 | / | / |  |
| Retroperitoneal region | 9 | 25.00 | / | / |  |
| Mediastinum | 14 | 38.89 | / | / |  |
| Other region | 2 | 5.56 | / | / |  |
| SD, standard deviation.  ^a^ Two-sided *χ^2^* test for distributions between neuroblastoma patients and controls | | | | | |
